# Absolute and non-invasive determination of the electron bunch length in a free electron laser using a bunch compressor monitor

**DOI:** 10.1038/s41598-024-56586-1

**Published:** 2024-03-15

**Authors:** Gian Luca Orlandi

**Affiliations:** Paul Scherrer Institut, Forschungsstrasse 111, Villigen PSI, 5232 Switzerland

**Keywords:** Driven linac FEL, Diagnostics, Electron bunch length, Temporal coherent radiation emission, Mathematics and computing, Physics, Techniques and instrumentation

## Abstract

In a linac driven Free Electron Laser (FEL), the shot-to-shot and non-invasive monitoring of the electron bunch length is normally ensured by Bunch Compressor Monitors (BCMs). The bunch-length dependent signal of a BCM results from the detection and integration—over a given frequency band—of the temporal coherent enhancement of the radiation spectral energy emitted by the electron beam while experiencing a longitudinal compression. In this work, we present a method that permits to express the relative variation of the bunch length as a function of the relative statistical fluctuations of the BCM and charge signals. Furthermore, in the case of a BCM equipped with two detectors simultaneously operating in two distinct wavelength bands, the method permits an absolute determination of the electron bunch length. The proposed method is beneficial to a FEL. Thanks to it, the machine compression feedback can be tuned against the absolute measurement of the bunch length rather than a bunch-length dependent signal. In a CW-superconducting-linac driven FEL, it can offer the precious opportunity to implement a fully non-invasive and absolute diagnostics of the bunch length.

## Introduction

The lasing performance of a linac driven Free Electron Laser (FEL) strongly relies on the stability over time of the low emittance and high density current features that the electron beam can achieve after the different acceleration and compression stages operated in the machine^1–12^. At every compression stage, the non-invasive and shot-sequential monitoring of the electron bunch length in a FEL can be ensured by Bunch Compressor Monitors (BCMs)^13–20^. The bunch-length dependent signal of a BCM results from the detection and integration over a given frequency band of the temporal coherent enhancement of the radiation spectral energy emitted by the electron beam. A temporal coherent enhancement of the radiation emission is observable in a FEL, for instance, while the electron beam is crossing the last dipole of a magnetic chicane^21–25^ or the hole of a diffraction radiation screen placed in a straight section just downstream^26–30^. Thanks to the non-invasiveness and the dependency—albeit not absolute—on the bunch-length, a BCM signal can be fruitfully exploited to feed back into the RF working point of the accelerator and stabilize the bunch compression during FEL operations. Three BCMs are in operation in SwissFEL^18,31^, two of them are integrated in the machine feedback. Aim of the present work is to show how the bunch-length dependent signal of a BCM can be suitably processed for the shot-to-shot tracking of the relative variation of the bunch length. Furthermore, in the case of a BCM equipped with two detectors simultaneously collecting and integrating the radiation pulse energy in two different wavelength bands, the method presented here allows for an absolute determination of the electron bunch length from the analysis of the relative statistical fluctuations of both the charge and BCM signals.

## Results

The results presented in this work are beneficial to the diagnostics of a particle accelerator and, in general, to the analysis of the radiation spectral energy emitted by relativistic electron beams at the threshold of the temporal coherent enhancement, i.e., at a wavelength comparable with the electron bunch length. In particular, they can find a general application in a linac driven FEL equipped with bunch length monitors, also named as bunch compressor monitors (BCMs). The BCM monitoring of the electron bunch length in a linac driven FEL results from the detection and fully integration—within a given wavelength band—of the radiation emitted by the electron beam at the end of a compression stage when, for instance, the electron beam crosses the last dipole of a magnetic chicane. The analysis of the BCM output signal hence allows for a shot-to-shot and non-invasive tracking of the electron bunch length variation. During normal machine operations, the tuning of the bunch compression settings can benefit from the bunch-length dependent signal of a BCM by means of a control loop feeding back into the field amplitude and phase of the accelerating structures.

Under steady state machine conditions, the BCM output signal experiences a shot-to-shot variation due to the statistical fluctuations of the bunch length and charge. The formal method described hereinafter permits to decode in the relative variation of the BCM signal the independent contributions of the statistical fluctuations of bunch length and charge. As below described, in the case of a BCM equipped with a single detector, the implementation of the proposed method permits a shot-sequential tracking of the relative variation of the electron bunch length as a function of the corresponding relative variations of the BCM and charge monitor signals. Furthermore, in the case of a BCM equipped with two different and independent detectors, the proposed method permits in addition the shot-sequential determination of the absolute value of the electron bunch length. As an explanatory example of the experimental context in which the proposed method can be implemented, we will refer to the case of the SwissFEL and of the related BCM instrumentation.

In SwissFEL^31,32^, a 28 ns long 2-bunch macro-pulse with a charge of 10 or 200 pC and a longitudinal length of about 3 ps (rms) is accelerated up to about 6 GeV at a repetition rate of 100 Hz and compressed down to about a few fs in two magnetic chicanes (BC1 and BC2). After BC2 and a further acceleration stage, a magnetic switch yard^33^ splits the 2 bunches off into the hard x-ray and soft x-ray brunches: ARAMIS^34^ and ATHOS^35^, respectively. A further bunch compression down to the sub-fs scale can be operated by means of a magnetic chicane (ECOL) just upstream of the ARAMIS undulator line. The three aforementioned magnetic chicanes (BC1, BC2 and ECOL) are equipped with dedicated BCMs. Both the BC1-BCM and ECOL-BCM detect the Edge Synchrotron Radiation (Edge-SR) from the front edge of the 4th dipole of the magnetic chicane^18^. Just downstream of BC2, a holed diffraction screen constitutes the spectral radiation source of the BCM.

The BC1-BCM^18^ being optimized for bunch lengths 220-290 fs (rms) is equipped with two broadband Schottky diodes with a sensitivity up to more than 2 THz. They are simultaneously illuminated by a SR light pulse that two beam-splitters in cascade split off into two distinct optical paths towards the two detectors. The two detectors are equipped with high-pass spectral THz filters with low-frequency cut-off of 0.3 and 0.6 THz, respectively. The BC2-BCM being designed for electron bunch length 3–25 fs (rms) is equipped with a Mercury Cadmium Telluride (MCT) detector with a sensitivity in the wavelength band 2–12 μm^18^. The ECOL-BCM—optimized for bunch length measurements in the range 0.7–3.0 fs (rms)^18^—is equipped with a pyrodetector and an optical fiber spectrometer covering the spectral wavelength band 0.9–4.0 μm and 0.9–2.5 μm, respectively. Both the pyrodetector and the spectrometer are illuminated by the same light pulse split apart in reflection and transmission by a calcium fluoride ($CaF_2$) beam splitter.

In Sect. “Methods”, we presents the details of a formal method that permits to express the shot-to-shot relative variations of the BCM signal—treated as a dependent output variable—as a function of the independent input variables of the relative variations of the bunch length and charge. In Sect. “Bunch length relative variation from a single detector BCM”, we will apply the proposed method to the case of a BCM equipped with a single detector. By means of numerical results, we will show how to perform a shot-to-shot tracking of the relative variation of the electron bunch length as a function of the corresponding relative fluctuation of the BCM and charge signals. Moreover, in the case of a BCM equipped with two independent detectors, in Sect. “Bunch length absolute determination from a two-detector BCM” we will present the results of a further implementation of the method for a shot-sequential absolute determination of the electron bunch length. Finally, a sensitivity study and numerical benchmarking of the method for different BCM response regimes under extreme temporal coherence/incoherence radiation emission conditions as well as under the effect of the electronic noise of the BCM detectors are presented in Sect. "Sensitivity studies of the method".

### Bunch length relative variation from a single detector BCM

In the case of a BCM equipped with a single detector integrating the radiation emitted by the electron beam in a given acceptance frequency band $\Delta \omega =(\omega _{max}-\omega _{min})$, the formal results described by Eqs. (10) and (11) permit to express the relative variation of the BCM signal $\frac{\Delta I}{I}$ with respect to a reference value—for instance, the mean value of the BCM signal readouts over a sequential acquisition of machine bunch shots—as a function of the corresponding relative variations of the bunch charge $\frac{\Delta N}{N}$ and of the electron bunch length $\frac{\Delta \sigma }{\sigma }$.

In the present context, it is worth observing that the study of the shot-sequential relative variation of the BCM signal versus the corresponding relative variation of the bunch length as well as of the charge can sound pleonastic. Indeed, by normalizing the measured BCM signal with respect to the square of the signal readout of a charge monitor—see for instance the BCM calibration work reported in^20^—the charge dependency of Eq. (3) on the number N of electrons in the bunch can be neutralized. Consequently, upon a suitable charge normalization of the BCM signal, the relative variation of the charge can be set to zero in Eq. (10), $\frac{\Delta N}{N}=0$. The so obtained formula in Eq. (10) hence simply correlates the relative variations of the BCM signal to those of the electron bunch length. Despite the clear advantages of a simplified mathematical formalism resulting from the charge normalization, in the present context we prefer to explicitly maintain in Eq. (10) the functional dependency on the relative charge variation $\frac{\Delta N}{N}$. This is for the sake of a formal completeness of the method description as well as to offer to possible users a complete modelling of the BCM response.

The measured quantities $\frac{\Delta I}{I}$ and $\frac{\Delta N}{N}$ result from the processing of the BCM and charge monitor signal readouts, respectively. They play the role of input variables in Eq. (10), whereas the variable $\frac{\Delta \sigma }{\sigma }$ is the unknown output variable. This output variable is modulated by the function *G*—see Eq. (11) - itself depending on a further unknown parameter, i.e., the absolute value of the electron bunch length $\sigma$. Provided that the reference BCM signal has been calibrated in advance by means of an absolute measurement of the electron bunch length $\sigma$—for instance, by means of a Transverse Deflecting Structure (TDS)^36–40^—Eqs.(10) and (11) allow for a shot-to-shot tracking of the relative variation of the bunch length $\frac{\Delta \sigma }{\sigma }$ as a function of the relative fluctuations of the BCM and charge monitor signals: $\frac{\Delta I}{I}$ and $\frac{\Delta N}{N}$.

**Figure 1:**
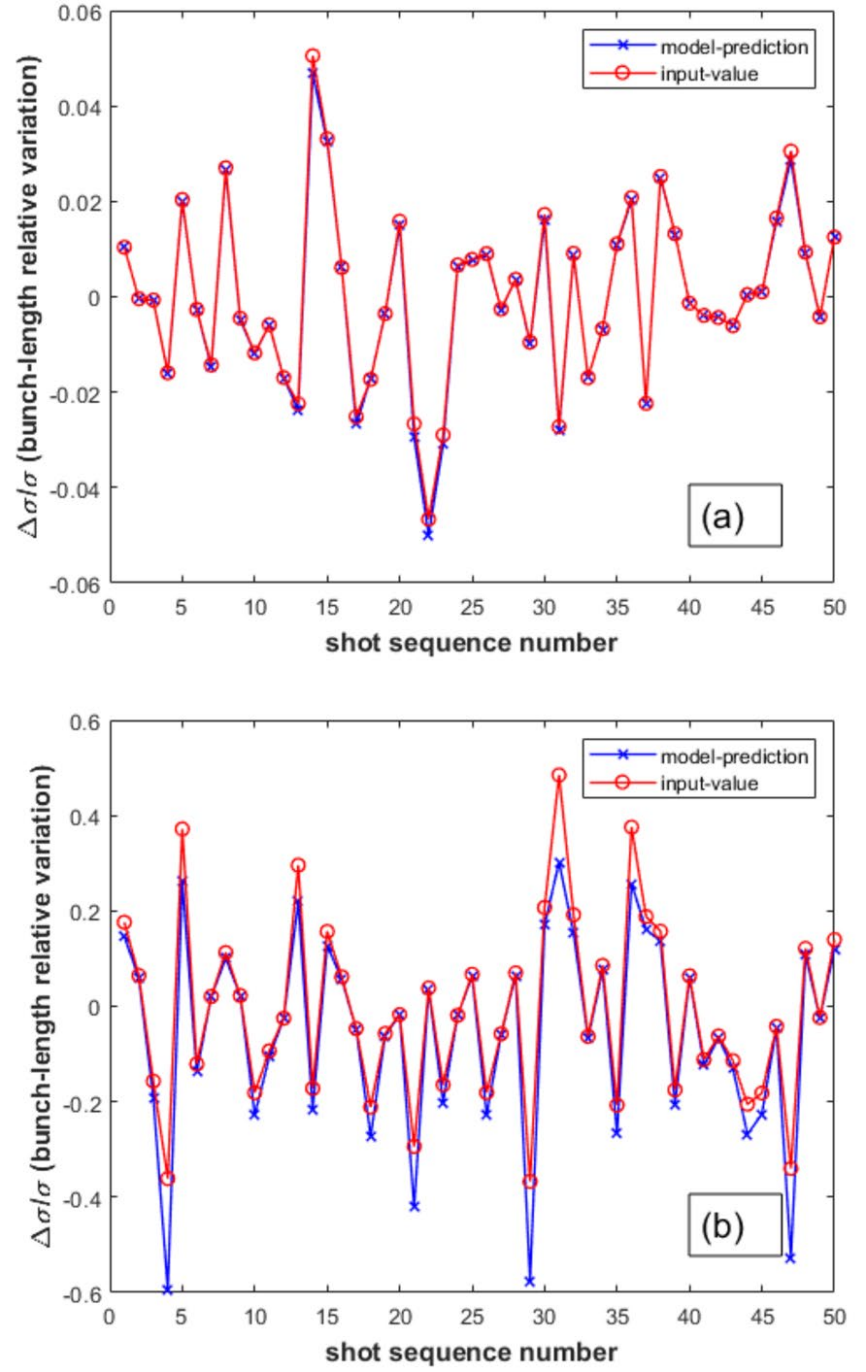
SwissFEL BC1-BCM ($0.3--2.0$ THz) and Gaussian FF with $\sigma =270$ fs: model-predicted $\frac{\Delta \sigma }{\sigma }$ vs model-input $\frac{\Delta \sigma }{\sigma }$ with rms deviations of 2$\%$ (**a**) and 20$\%$ (**b**); rms deviation of 1$\%$ for $\frac{\Delta N}{N}$.

**Figure 2:**
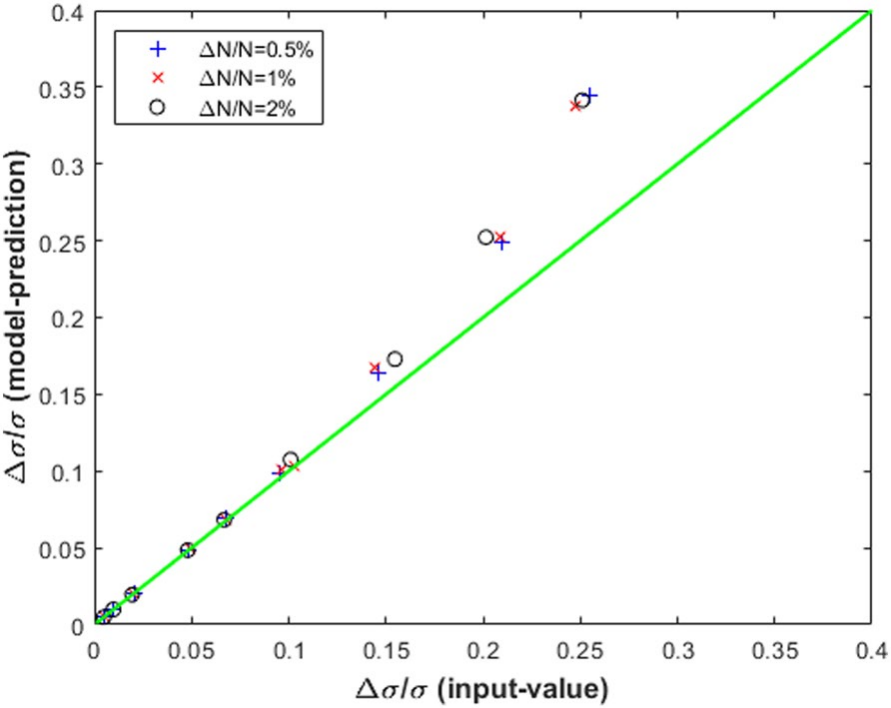
Standard deviations of model-predicted $\frac{\Delta \sigma }{\sigma }$ vs model-input reference values for $\frac{\Delta N}{N}$ with rms deviations 0.5–2$\%$. See also caption Fig. 1.

In the case of the SwissFEL BC1-BCM with an acceptance frequency band of $0.3--2.0$ THz, the capability of the Eqs. (10) and (11) to predict $\frac{\Delta \sigma }{\sigma }$ was numerically tested for a Gaussian form-factor (FF) with $\sigma =270$ fs, see Fig. 1. A sequence of 500 relative variations of the bunch length $\frac{\Delta \sigma }{\sigma }$ around the reference value was randomly extracted according to a normally distributed generator with rms deviations from 0.5 up to 25$\%$. With the same random extraction procedure, a sequence of 500 relative variations of the beam charge $\frac{\Delta N}{N}$ was also obtained for rms deviations $0.5\%$, $1\%$ and $2\%$. From the randomly extracted sequences of $\frac{\Delta \sigma }{\sigma }$, the corresponding relative variation $\frac{\Delta I}{I}$ of the BCM signal was obtained via Eqs. (3), (4 and (5). The so obtained sequences of $\frac{\Delta N}{N}$ and $\frac{\Delta I}{I}$ were used as input variables of the Eqs. (10) and (11) to evaluate the relative variation of the bunch length $\frac{\Delta \sigma }{\sigma }$. In Fig. 1, a sequence of model-predicted values of $\frac{\Delta \sigma }{\sigma }$—Eqs. (10) and (11)—is compared with homologous model-input sequences randomly generated with rms deviations 2$\%$ and 20$\%$ for a charge fluctuation sequence $\frac{\Delta N}{N}$ with rms deviation of 1$\%$. In Fig. 2, the standard deviations of the 500-shot sequences of the model-predicted relative variations of the electron bunch length $\frac{\Delta \sigma }{\sigma }$ are plotted versus the corresponding model-input values for different rms deviations of $\frac{\Delta N}{N}$. The model-predicted and model-input sequences of $\frac{\Delta \sigma }{\sigma }$ remain reasonably aligned up to a rms deviation of 10$\%$, see Fig. 2.

The absolute determination of the electron bunch length from the analysis of a BCM signal is a result already achieved in a linac driven FEL. In the European XFEL^20^, for instance, the calibration of a BCM signal by means of a TDS measurement allows for the estimate of the electron bunch length from a lookup table of the BCM signal output levels. The determination of a calibration curve of the BCM response vs TDS measurement as described in^20^ is an efficacious experimental approach that can get rid of possible bunch-length independent effects affecting the BCM signal response such as, for instance, a low frequency cut-off of the single particle spectrum or a not uniform transfer function of the detector response. Novelty of the formal method proposed in the present work stays instead in the attempt to go beyond a phenomenological approach to the BCM calibration. Aim of the present work is to establish a mathematical formalism expressing the relative statistical fluctuations of a BCM signal as a function of the corresponding relative variations of the electron bunch length—and charge as well. As better described below, this innovative mathematical approach should offer a change of perspective in the analysis of the statistical fluctuations of the BCM—and charge - signals as well as a new practical tool for determining the absolute value of the electron bunch length from the processing of the BCM signals.

### Bunch length absolute determination from a two-detector BCM

In the previous subsection, we considered the case of a BCM equipped with a single detector. We showed how from Eqs. (10) and (11) it is possible to track the relative variation of the electron bunch length $\frac{\Delta \sigma }{\sigma }$ with respect to a reference value—for instance the mean value over a shot-sequential acquisition of bunch signal under steady state machine operations—as a function of the corresponding relative variations of the BCM and charge monitor output signals $\frac{\Delta I}{I}$ and $\frac{\Delta N}{N}$, respectively.

We consider now the case of a BCM equipped with two independent detectors. They are supposed to be simultaneously illuminated by the same radiation pulse split off into two distinct optical paths by a beam splitter. They are also supposed to perform the full integration of the radiation energy spectrum over two different wavelength bands $\Delta \omega _{i}=(\omega _{max}^{i}-\omega _{min}^{i})$ with $i=1,2$. According to Eq. (12), from the processing of the two BCM-detectors and charge monitor signals—$\left( \frac{\Delta I}{I}\right) _i$ and charge $\frac{\Delta N}{N}$, respectively—an absolute determination of the electron bunch length can be obtained.

**Figure 3:**
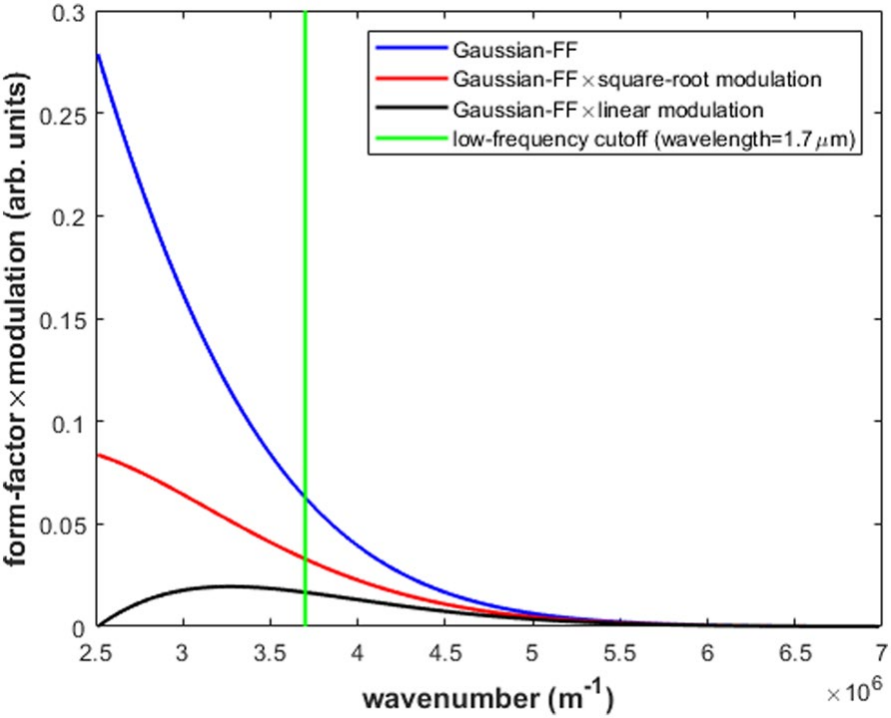
Pure, square-root and the linear modulated Gaussian-FF [$\sigma =1.5$ fs (rms)] in the wavelength band 0.9–2.5 μm of the ECOL spectrometer. The vertical line marks the wavelength band 0.9–1.7 μm [wavenumber=$(3.7--7.0)\times 10^6 m^{-1}]$, see also Fig. 5.

The capability of Eq. (12) to predict the absolute value of the bunch length $\sigma$ was numerically tested in relation to the ECOL-BCM of SwissFEL for a Gaussian FF with $\sigma$ ranging from 0.5 to 3.0 fs, step 0.5 fs. We supposed a uniform transfer function for the ECOL-BCM pyrodetector in the wavelength band 0.9–4.0 μm. Whereas, for the spectrometer, we supposed a transfer function either uniform or affected by a low-frequency modulation cutoff $M(\omega )$ in the wavelength bands 0.9–2.5 μm and 0.9–1.7 μm, respectively, see Fig. 3. In particular, we considered a square-root ($\alpha =0.5$, $M_{min}=0.3$) and a linear ($\alpha =1.0$, $M_{min}=0.0$) modulation function of the spectrometer response:

1 $$\begin{aligned} M(\omega )=\left( \frac{1-M_{min}}{\omega _{max}^\alpha -\omega _{min}^\alpha }\right) \omega ^\alpha +\frac{\omega _{max}^\alpha M_{min}-\omega _{min}^\alpha }{\omega _{max}^\alpha -\omega _{min}^\alpha }. \end{aligned}$$

In Fig. 3, the pure, square-root and the linear modulated Gaussian FF are plotted for $\sigma =1.5$ fs. For each value of $\sigma$ considered in the numerical simulation, relative statistical fluctuations of the two ECOL-BCM signals $\left( \frac{\Delta I}{I}\right) _i$—with $i=1,2$—were calculated from randomly extracted sequences of $\frac{\Delta \sigma }{\sigma }$ with rms deviation 1$\%$. Similarly, sequences of $\frac{\Delta N}{N}$ with rms deviation of 1$\%$ were randomly extracted.

**Figure 4:**
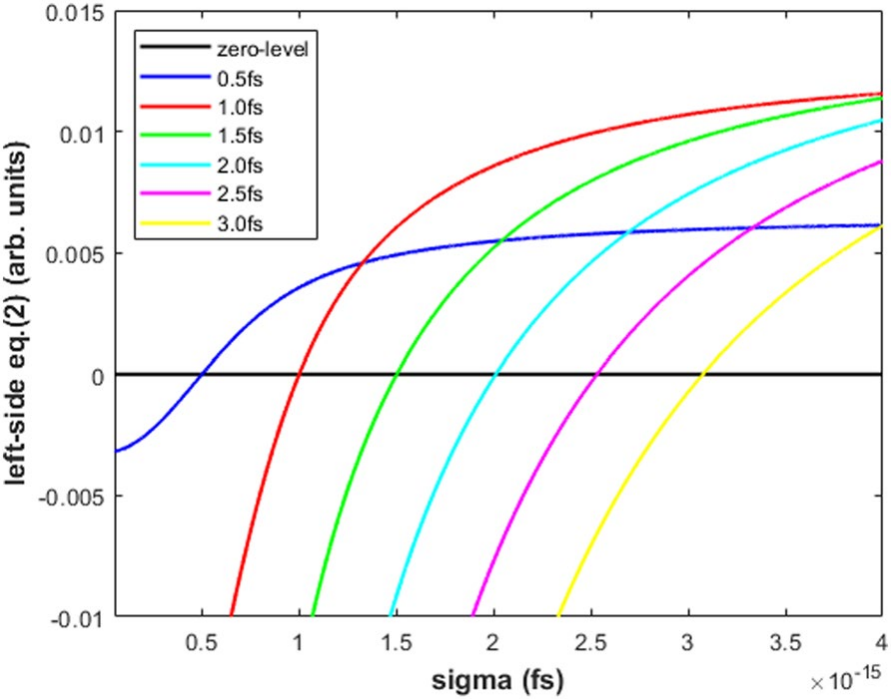
Test results of Eq. (2) from the SwissFEL ECOL-BCM: the abscissae of the curve intercepts with the “zeros-level” line are the model-estimated absolute values of $\sigma$. Simulation settings: pyrodetector and spectrometer with wavelength bands 0.9–4.0 μm and 0.9–2.5 μm, respectively; Gaussian FF with $\sigma$=0.5–3.0 fs; model-input $\frac{\Delta \sigma }{\sigma }$ and $\frac{\Delta N}{N}$ with rms deviation of 1 $\%$.

**Figure 5:**
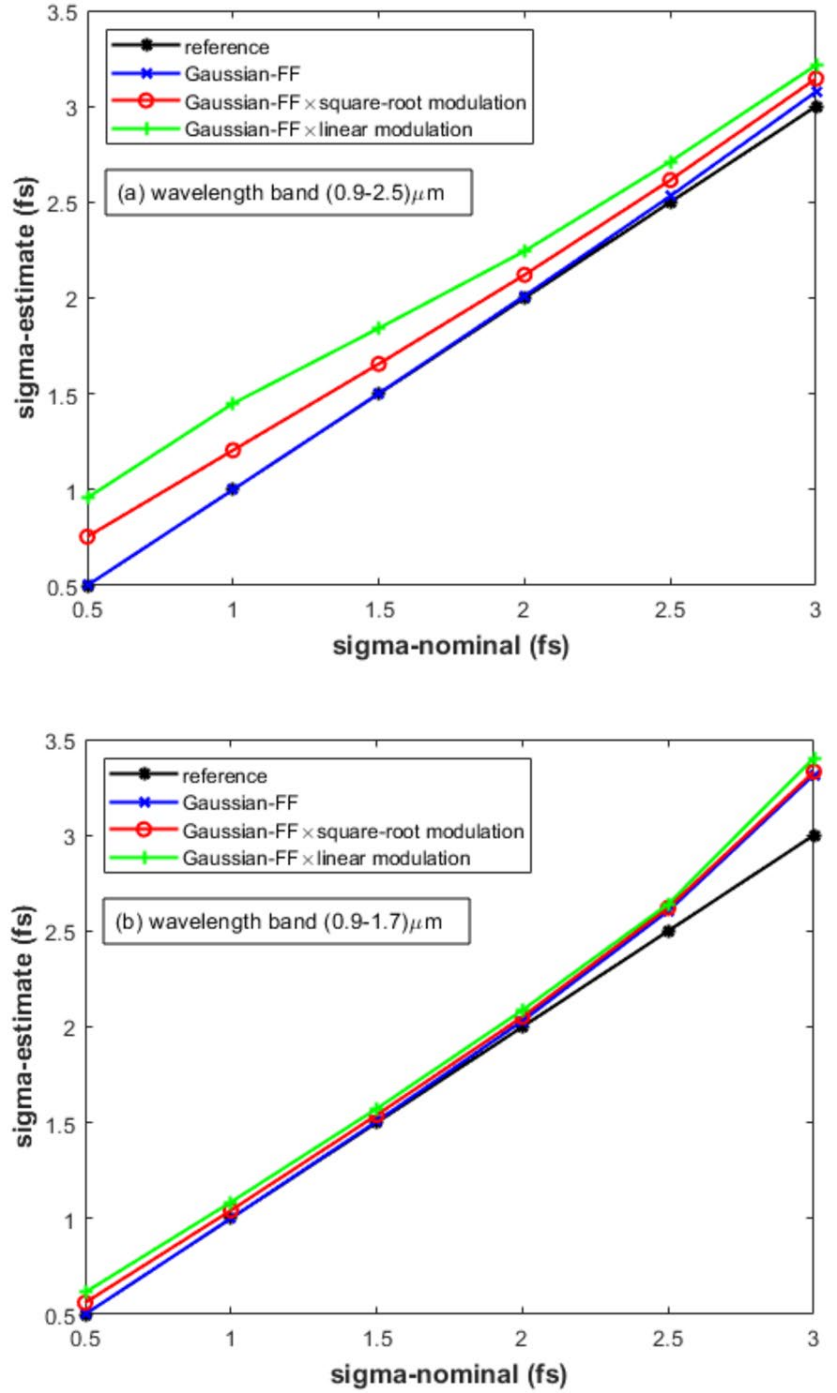
Test results of Eq. (2) from the SwissFEL ECOL-BCM: model-estimated vs model-input values of $\sigma$. Simulation settings: Gaussian FF with $\sigma$=0.5–3.0 fs filtered by a uniform, square-root and linear modulated spectrometer response in the wavelength bands (**a**) 0.9–2.5 μm and (**b**) 0.9–1.7 μm; model-input sequences of $\frac{\Delta \sigma }{\sigma }$ and $\frac{\Delta N}{N}$ with rms deviation of 1 $\%$. The “blue” curve in Fig. 5**a**—Gaussian FF—draws the abscissae of the curve intercepts to the “zero-level” as shown in Fig. 4.

The so obtained sequences of $\left( \frac{\Delta I}{I}\right) _i$ - with $i=1,2$—and $\frac{\Delta N}{N}$ were used as input variables of Eq. (12). The complex functional dependence of Eq. (12) prevents a straightforward inversion of the formula with respect to $\sigma$. Under steady state machine operations, an easy way to handle Eq. (12) and determine $\sigma$ consists in: (a) applying a standard deviation operator to the Eq. (12) when running over the measured sequences of relative variations of BCM signals $\left( \frac{\Delta I}{I}\right) _i$ (with $i=1,2$) and charge monitor readout $\frac{\Delta N}{N}$; finally, (b) finding the “zeros” of the resulting equation as a function of the unknown parameter $\sigma$:

2 $$\begin{aligned}{} & {} \setminus std\left( \left[ \left( \frac{\Delta I}{I}\right) _2-2\frac{\Delta N}{N}\right] \right)- \nonumber \\{} & {} +\setminus abs\left( \frac{G(\sigma ,(\Delta \omega )_2)}{[G(\sigma ,(\Delta \omega )_2)-G(\sigma ,(\Delta \omega )_1)]}\right) \nonumber \\{} & {} \times \setminus std\left( \left[ \left( \frac{\Delta I}{I}\right) _2-\left( \frac{\Delta I}{I}\right) _1\right] \right) =0 \end{aligned}$$

where the symbols $\setminus std$ and $\setminus abs$ in the previous equation are indicating the operations of standard deviation and absolute value, respectively. It is worth noting that the arguments of the operator standard deviation $\setminus std$ in Eq. (2) are adimensional and consequently can be added up. According to the aforementioned approach, we handled by means of Eq. (2) the processing of Eq. (12) running over the input data $\left( \frac{\Delta I}{I}\right) _i$—with $i=1,2$—and $\frac{\Delta N}{N}$. We finally obtained the absolute values of the bunch length by calculating the“zeros” of Eq. (2) as a function of the test variable $\sigma$ as shown in Fig. 4, where the case of a pure Gaussian FF over the full wavelength band (0.9–2.5 μm) of the ECOL spectrometer was considered. The abscissae of the curve intercepts with the “zeros” level in Fig. 4 permit to determine—via Eq. (2)—the electron bunch length within an error from 0.5$\%$ to 2.5$\%$, see “blue” curve (“Gaussian FF”) in Fig. 5a compared to the “black” curve (“reference”).

For the aforementioned case of the ECOL-BCM, under the hypothesis of a pure, square-root and linear frequency-modulated Gaussian FF—see Fig. 3—in the spectrometer wavelength bands 0.9–2.5 μm and 0.9-1.7 μm, the complete overview of the absolute determination of the bunch length $\sigma$ based on the “zeros” calculation of Eq. (2) is shown in Fig. 5 for model-input values of $\sigma$ in the range 0.5–3.0 fs. With the exclusion of the less significative values 0.5 and 3.0 fs—the ECOL-BCM design is indeed optimized for the bunch length interval 0.7–3.0 fs^18^—the error on the model-predicted $\sigma$ is in the range 20-5$\%$ and 5-3$\%$ in the case of a square-root frequency modulation of the Gaussian FF in the spectrometer wavelength bands 0.9–2.5 μm and 0.9–1.7 μm, respectively, see Fig. 5. Whereas, in the case of a linear modulation of the Gaussian FF in the spectrometer wavelength band 0.9–1.7 μm, the error on the model-predicted $\sigma$ stays in the range 8-4$\%$, see Fig. 5b. In conclusion, due to the features of Eqs. (2) and (12) to process the relative variations of the input variables $\left( \frac{\Delta I}{I}\right) _i$—with $i=1,2$—and $\frac{\Delta N}{N}$, the estimate of the absolute value of the bunch length $\sigma$ is characterized by robustness and weakly dependency on unexpected or unknown bunch-length independent frequency modulation of the FF, see Eqs. (3) and (4).

## Methods

The case of an electron beam emitting synchrotron radiation when crossing the last dipole of a magnetic chicane or diffraction radiation when crossing the hole of a metallic coated screen will be considered hereinafter. In particular, we will consider the formal expression of the radiation energy spectrum emitted by the electron beam as it can be observed by a detector with an acceptance wavelength band matching with the temporal coherent enhancement of the radiation emission. Upon integration over the acceptance solid angle $\Delta \Omega$ of the BCM detector, the spectral distribution of the radiation energy emitted per unit of angular frequency $\omega =2\pi \nu$ by a bunch of N electrons at the temporal coherent threshold of the N-quadratic enhancement reads (for highly collimated beams):

3 $$\begin{aligned} \frac{dI^{Ne}(\omega )}{d\omega }\simeq N(N-1)F(\omega )\frac{dI^e(\omega )}{d\omega }\simeq N^2F(\omega )\frac{dI^e(\omega )}{d\omega }, \end{aligned}$$

where $\frac{dI^e(\omega )}{d\omega }$ is the single particle energy spectrum and $F(\omega )$ is the longitudinal form factor (FF) of the electron beam^41,42^.

The approximation of the bunch form factor to the simple longitudinal contribution as defined in the following Eq. (4) can be justified by the highly collimation (low transverse emittance) characterizing the electron beam in a linac driven FEL. The shot-to-shot variation of the bunch charge may have an influence on both the longitudinal and transverse dimensions of the electron beam. Indeed, space charge and wakefield effects can feed back into the beam transverse and longitudinal sizes of the electron beam as a function of the shot-to-shot variation of the beam charge. A modelling of the charge induced variations of the electron bunch length is far from the aim of the present work. Nevertheless, the method of analysis of the BCM signal that we are proposing states a formal correlation among the relative statistical fluctuations of the bunch charge, bunch length and BCM signals. The proposed method itself offers the opportunity to study the effect on the beam dynamics of the shot-to-shot fluctuations of the beam charge and to correlate them with the related variations of the bunch longitudinal length. About the shot-sequential variations of the transverse profile of the beam due to possible space-charge induced variations of the normalized emittance, we can reasonably assume that their effect on the radiation energy spectrum and consequently on the BCM signal can be considered negligible thanks to the highly collimation feature characterizing the electron beams in a linac driven FEL.

For a Gaussian beam with a length (standard deviation) $\sigma$, the FF being defined as the square module of the Fourier transform of the density distribution function ρ_z_(z) of the N electron coordinates along the longitudinal direction reads:

4 $$\begin{aligned} F(\omega )=\left| \int _{-\infty }^{+\infty }e^{j\omega z/c}\rho _z(z)dz\right| ^2=e^{-(\frac{\omega \sigma }{c})^2}. \end{aligned}$$

N.B.: with the exception of the present Sect. “Methods”, where the bunch length is correctly expressed in a length-unit, in the rest of the manuscript—for the sake of simplicity—the bunch-length will be referred in terms of a time-unit (fs), i.e., the normalization $\sigma /c$ will be implicitly supposed.

Next, we will assume that the single electron energy spectrum $\frac{dI^e(\omega )}{d\omega }$ is either slowly dependent on or even independent of the frequency in the considered frequency band. Such an assumption is reasonably valid in the case of the two Edge-SR based BCMs of SwissFEL^43,44^. Moreover, the proposed method, being based on the processing of the relative statistical fluctuation of the BCM with respect to a reference value, is mainly sensitive to the bunch length dependent component of Eq. (3) rather than to those parameters which in Eq. (3) are bunch-length invariant in the given frequency band such as, for instance, a low frequency diffractive cutoff of the single particle radiation energy spectrum or the frequency response function of the detector. Therefore, possible bunch-length invariant factors affecting the radiation energy spectrum tend to be smoothed down. According to such a hypothesis, let’s normalize the formula in Eq. (3) with respect to $\frac{dI^e(\omega )}{d\omega }$. Afterwards, let’s calculate in both sides of the resultant normalized expression of Eq. (3) the integral over the acceptance frequency band $\Delta \omega =(\omega _{max}-\omega _{min})$ of the detector and call *I* the integral of the “normalized” radiation energy spectrum of the electron bunch which corresponds to a given form factor $F(\omega )$:

5 $$\begin{aligned} I=\int _{\omega _{min}}^{\omega _{max}}d\omega \left( \frac{dI^{Ne}(\omega )}{d\omega }/\frac{dI^e(\omega )}{d\omega }\right) . \end{aligned}$$

Upon applying the natural logarithm to both sides of the so obtained normalized and integrated expression of Eq. (3), we obtain the following equation:

6 $$\begin{aligned} \ln (I)=2\ln (N)+\ln (\int _{\omega _{min}}^{\omega _{max}}d\omega F(\omega )), \end{aligned}$$

By differentiating Eq. (6) and converting the infinitesimal differentials of the quantities in Eq. (6) into finite differences calculated over the time unit of the machine repetition rate, it is possible to express the shot-to-shot relative variation of the BCM signal $\frac{\Delta I}{I}=\frac{I^*-I}{I}$ as a function of the corresponding relative fluctuation of the longitudinal form factor $\frac{\Delta F}{F}=\frac{F^*-F}{F}$—i.e., of the electron bunch length $\frac{\Delta \sigma }{\sigma }=\frac{\sigma ^*-\sigma }{\sigma }$—and of the relative fluctuation of the number of electrons $\frac{\Delta N}{N}=\frac{N^*-N}{N}$ in the bunch:

7 $$\begin{aligned} \frac{\Delta I}{I}=2\frac{\Delta N}{N}+\frac{\int _{\omega _{min}}^{\omega _{max}}d\omega [F^*(\omega )-F(\omega )]}{\int _{\omega _{min}}^{\omega _{max}}d\omega F(\omega )}. \end{aligned}$$

Thanks to a Taylor series expansion at the first order in $\frac{\Delta \sigma }{\sigma }$, the FF variation in the integrand of Eq. (7) can be explicitly expressed as^45^

8 $$\begin{aligned}{} & {} F^*(\omega )-F(\omega )=e^{-(\frac{\omega \sigma }{c})^2(1+\frac{\Delta \sigma }{\sigma })^2}-e^{-(\frac{\omega \sigma }{c})^2}\nonumber \\{} & {} \simeq -2\frac{\Delta \sigma }{\sigma }\left( \frac{\omega \sigma }{c}\right) ^2e^{-(\frac{\omega \sigma }{c})^2}=-2\frac{\Delta \sigma }{\sigma }\left( \frac{\omega \sigma }{c}\right) ^2F(\omega ) \end{aligned}$$

and the integral of the FF in Eq. (7) explicitly calculated

9 $$\begin{aligned}{} & {} \frac{\int _{\omega _{min}}^{\omega _{max}}d\omega [F^*(\omega )-F(\omega )]}{\int _{\omega _{min}}^{\omega _{max}}d\omega F(\omega )}\nonumber \\{} & {} \simeq -2\frac{\Delta \sigma }{\sigma }\frac{\int _{\omega _{min}}^{\omega _{max}}d\omega \left( \frac{\omega \sigma }{c}\right) ^2e^{-(\frac{\omega \sigma }{c})^2}}{\int _{\omega _{min}}^{\omega _{max}}d\omega e^{-(\frac{\omega \sigma }{c})^2}}\nonumber \\{} & {} =-2\frac{\Delta \sigma }{\sigma }\frac{\left[ \frac{-2\omega \sigma e^{-(\frac{\omega \sigma }{c})^2}+\sqrt{\pi }c\, erf\left( \omega \sigma /c\right) }{4\sigma } \right] _{\omega _{min}}^{\omega _{max}}}{\left[ \frac{\sqrt{\pi }c}{2\sigma }erf(\omega \sigma /c)\right] _{\omega _{min}}^{\omega _{max}}}, \end{aligned}$$

where erf(x) is indicating the error function^46^. In conclusion, from Eqs. (7), (8) and (9) the relative fluctuation of the BCM signal $\frac{\Delta I}{I}$ with respect to a reference value—for instance, the mean value over a temporal sequence of acquisitions—can be expressed as a function of the corresponding relative variations of the number of electrons in the bunch $\frac{\Delta N}{N}$ and of the electron bunch length $\frac{\Delta \sigma }{\sigma }$:

10 $$\begin{aligned} \frac{\Delta I}{I}=2\frac{\Delta N}{N}+\frac{\Delta \sigma }{\sigma }G(\sigma ,\Delta \omega ), \end{aligned}$$

where

11 $$\begin{aligned} G(\sigma ,\Delta \omega )=\left\{ \frac{2\sigma }{\sqrt{\pi }c}\frac{\left[ e^{-(\frac{\omega \sigma }{c})^2}\omega \right] _{\omega _{min}}^{\omega _{max}}}{\left[ erf(\omega \sigma /c)\right] _{\omega _{min}}^{\omega _{max}}}-1\right\} . \end{aligned}$$

According to Eqs. (10) and (11), the simultaneous determination of $\sigma$ and $\frac{\Delta \sigma }{\sigma }$ seems to be not straightforward from the analysis of the signal of a BCM equipped with a single detector. Nevertheless, provided that an absolute calibration of the BCM can be carried out by means of an independent and absolute monitor—for instance, a Transverse Deflecting Structure (TDS)^36–40^ - Eqs. (10) and (11) permit to perform a shot-to-shot tracking of the relative variation of the bunch length $\frac{\Delta \sigma }{\sigma }$ as a function of the relative fluctuations of the BCM and charge monitor signals: $\frac{\Delta I}{I}$ and $\frac{\Delta N}{N}$.

For a BCM equipped with two independent detectors simultaneously integrating in two distinct frequency bands $\Delta \omega _{i}=(\omega _{max}^{i}-\omega _{min}^{i})$—with $i=1,2$—the same radiation pulse split off by a beam splitter - $\left( \frac{\Delta I}{I}\right) _i$—an absolute determination of the electron bunch length $\sigma$ is instead possible via Eqs. (10) and (11) by means of the following formula:

12 $$\begin{aligned} \left[ \left( \frac{\Delta I}{I}\right) _2-2\frac{\Delta N}{N}\right] ={} & {} \frac{G(\sigma ,(\Delta \omega )_2)}{[G(\sigma ,(\Delta \omega )_2)-G(\sigma ,(\Delta \omega )_1)]}\nonumber \\{} & {} \times \left[ \left( \frac{\Delta I}{I}\right) _2-\left( \frac{\Delta I}{I}\right) _1\right] . \end{aligned}$$

Despite the complex functional dependency on the electron bunch length $\sigma$, the formula in Eq. (12) permits to track the variation of the absolute value of the electron bunch length, - from a machine shot to a next one—as a function of the synchronously measured relative variations of the two BCM detectors and charge monitor signals: $\left( \frac{\Delta I}{I}\right) _i$ (with $i=1,2$) and $\frac{\Delta N}{N}$, respectively. Such a sequential determination of the absolute value of bunch length can be in practice obtained by calculating the “zeros” of the formula Eq. (12) as a function of a test variable $\sigma$. In other words, for a given sequence of synchronously acquired BCM and charge monitor signals, the shot-to-shot variation of the absolute value of the electron bunch length can be determined by running the unknown parameter $\sigma$ over a test interval until a suitable value of $\sigma$ allowing for a correct equalization of the two sides of the Eq. (12) has been identified. In the Sect. “Results”, numerical results obtained under the hypothesis of steady state machine operations are presented.

As already stated, the method of signal analysis proposed in this manuscript permits to enlarge the potentialities of the BCMs with the further option to predict the absolute value of the electron bunch length. Despite the very basic operation mode consisting in the full integration of the radiation energy spectrum over the wavelength band of acceptance of the detector, a BCM—if equipped with two independent detectors—can compete with other spectroscopy-based diagnostics of the electron bunch length, at least, for the determination of the absolute value of the bunch length. In this sense, the proposed method of analysis of the BCM signals can be considered as a valid complement to broad-band spectroscopy techniques of Fourier analysis of the radiation energy spectrum aiming at the reconstruction of the current profile of the electron beam^19,47–56^.

## Sensitivity studies of the method

It is worth evaluating the sensitivity of the proposed method for the absolute determination of the electron bunch length when, as a function of the electron bunch length, the effective response of the BCM shifts—over the wavelength band of acceptance of the BCM detectors—from the transition region of the temporal coherent threshold of the radiation energy emission towards the fully temporal coherent or incoherent regions. Indeed, the proposed mathematical formalism has been developed under the implicit hypothesis that, for a given electron bunch, the onset of the charge-quadratic temporal coherent enhancement of the radiation energy spectrum matches with the optimal frequency response of the BCM detectors. Moreover, in the numerical simulations presented for the benchmark of the proposed method, the role played by the electronic noise of the detector was not taken into consideration. In the present section, we will present a sensitivity study of the model predictions as a function of the electron bunch length so that the effective BCM response can span the regimes from a dominant temporal coherence to a dominant temporal incoherence. In addition, in the sensitivity study of the model prediction of the bunch-length, we will also simulate the effect of the electronic noise of the detectors. In this study, we will consider the case of the ECOL-BCM which has a bunch length sensitivity between $0.7--3.0$ fs (rms).

**Figure 6:**
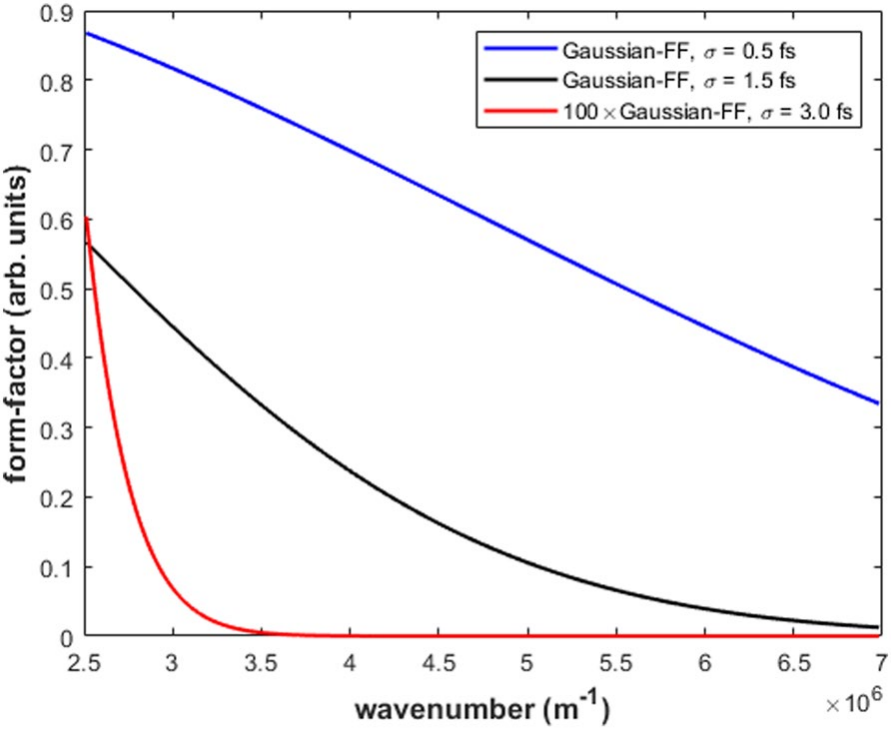
Plots of Gaussian form-factors calculated for different bunch-lengths ($\sigma =$ 0.5, 1.5 and 3.0 fs, rms) as a function of the wavenumber ($2\pi /\lambda$) with the wavelength $\lambda$ running over the wavelength band of acceptance of the ECOL-BCM spectrometer (0.9–2.5) μm.

In Fig. 6, the numerical simulations of Gaussian form-factors corresponding to bunch length of 0.5, 1.5 and 3.0 fs (rms) are shown in the acceptance wavelength band of the ECOL-BCM spectrometer. It should be noted that the form factor corresponding to 3.0 fs (rms) has been multiplied by a factor 100 to render visible in the plot the onset of the temporal coherent threshold. With reference to the plots in Fig. 6, in the case of the ECOL-BCM, we can reasonably suppose that the detector response drifts into a regime of a dominant temporal incoherence for a bunch-length equal to or larger than 3.0 fs (rms); whereas, for a bunch length less than 0.7 (rms), the detector response stays in a regime of a dominant temporal coherence.

The numerical simulation of the effect of the electronic noise of the BCM detectors requires a preliminary modelling assumption. If the electronic noise of the detector is supposed to be a fraction of the detector signal baseline under a regime of fully temporal incoherence and if this is supposed simply to add up to the total detector signal (i.e., the sum of the temporal incoherent and coherent contributions of the radiation energy spectrum), the electronic noise of the detector has no effect on the model prediction of the electron bunch. In this case, even for very unrealistic values of the signal-to-noise ratio, we checked that the electronic noise has no effect on the model prediction of the bunch-length whatever is the bunch length in the sensitivity region of the ECOL-BCM. The effect of the electronic noise of the detector starts playing an observable effect on the model prediction of the electron bunch length if the detector electronic noise is supposed to be an additive fraction of the total detector signal (sum of the temporal coherent and incoherent contributions). In the following we will suppose that both the two detectors of the ECOL-BCM experience simultaneously the same percentage level of electronic noise. We performed the numerical simulations for a percentage ratio of the detector electronic noise to signal of $0.5\%$ and $1.0\%$ (rms).

**Figure 7:**
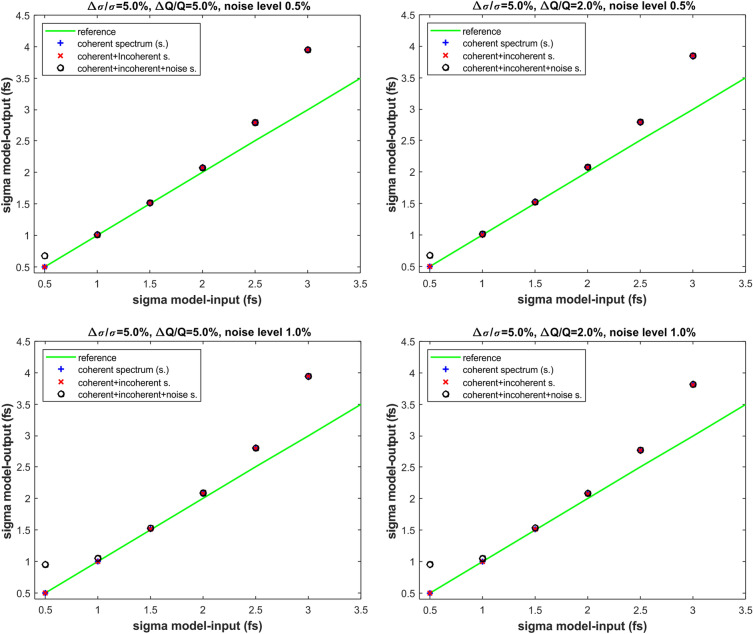
Model-prediction versus model-input of the electron bunch-length $\sigma$ under the hypothesis to consider the three different scenarios for the BCM detector signal response: “coherent spectrum”, “coherent+incoherent spectrum”, “coherent+incoherent+noise spectrum”. Numerical simulations performed for relative statistical fluctuations of the bunch-length $\frac{\Delta \sigma }{\sigma }=5\%$, of the charge of $\frac{\Delta N}{N}=2\%$ and $5\%$ and of the ratio-noise-to-signal=$0.5\%$ and $1.0\%$.

**Figure 8:**
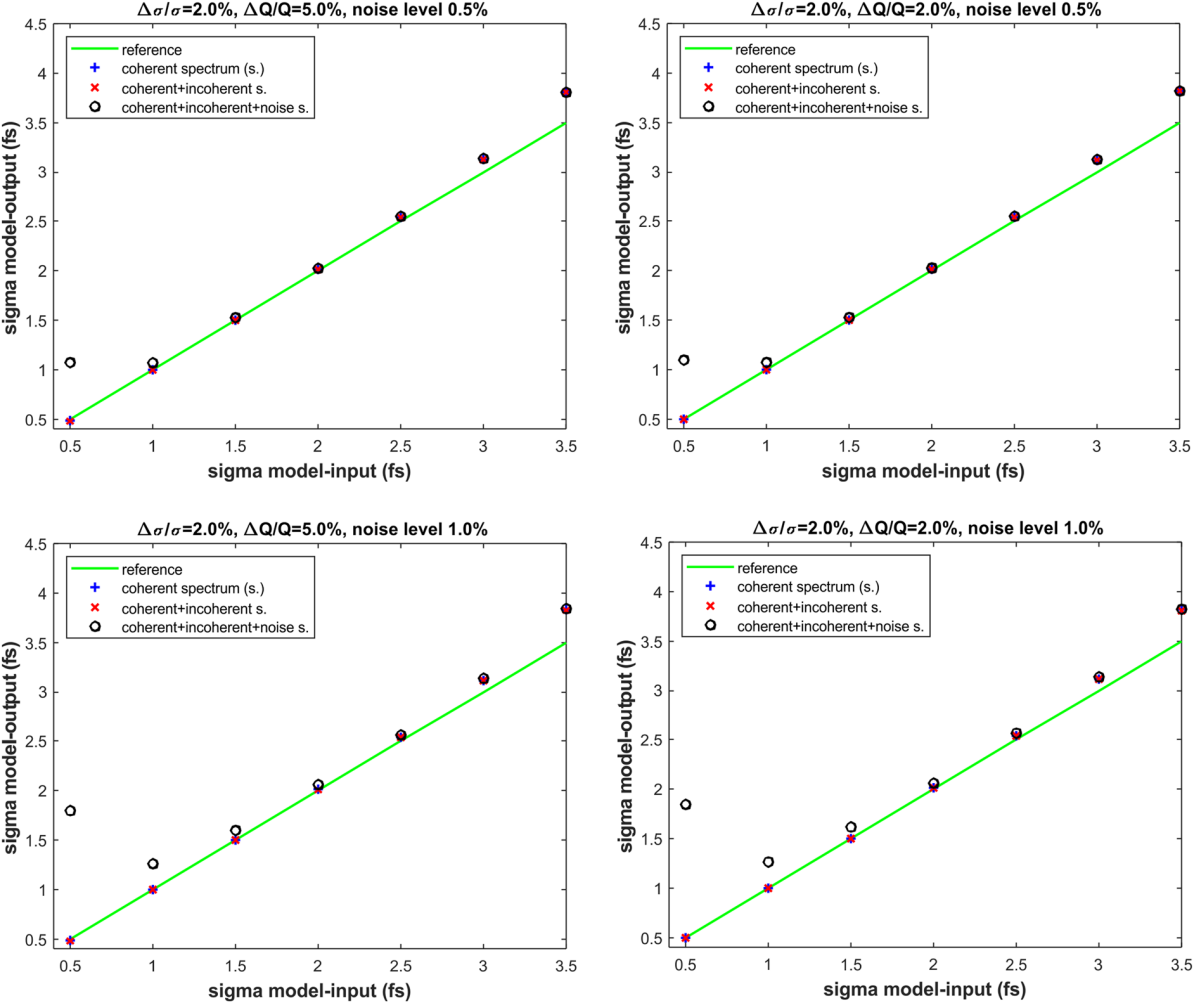
Model-prediction versus model-input of the electron bunch-length $\sigma$ under the hypothesis to consider the three different scenarios for the BCM detector signal response: “coherent spectrum”, “coherent+incoherent spectrum”, “coherent+incoherent+noise spectrum”. Numerical simulations performed for relative statistical fluctuations of the bunch-length $\frac{\Delta \sigma }{\sigma }=2\%$, of the charge of $\frac{\Delta N}{N}=2\%$ and $5\%$ and of the ratio-noise-to-signal=$0.5\%$ and $1.0\%$.

Under this hypothesis, we obtained the results of the numerical simulations plotted in Figs. 7 and 8. The results in Figs. 7 and 8 represent a benchmark of the formula given in Eq. (2) under the three following scenarios for the signal response of the two detectors of the ECOL-BCM. In Figs. 7 and 8 the label “coherent spectrum” is indicating that the signal response of the two detectors of the BCM-ECOL has been numerically simulated exactly according to Eq. (3) and related Eqs. (4) and (5). The label “coherent+incoherent spectrum” is indicating that the ECOL-BCM signals results from the formulae in Eqs. (3), (4) and (5) with the suitable addition of the linear contribution of the beam charge to the radiation energy spectrum. Whereas the label “coherent+incoherent+noise spectrum” is indicating the scenario of the further addition of the electronic noise to the detector signal of the previous case.

From the analysis of the results in Figs. 7 and 8, we first observe that, with the exclusion of the extreme values—0.7 and 3.0 fs—of the interval of bunch-length sensitivity of the ECOL-BCM, the model prediction of the bunch length matches very well with the model input values of the bunch-length for all the three scenarios of signal response we considered for the ECOL-BCM. For a bunch length of 2.5 fs—i.e., under a so called dominant temporal-incoherence regime—and a relative statistical fluctuation of the bunch-length $\frac{\Delta \sigma }{\sigma }$ of $5\%$ (rms), the maximum deviation between model-input and model-output values is about 12$\%$.

The effect of the relative statistical fluctuations of the bunch charge $\frac{\Delta N}{N}$ seems to be much smaller and only slightly observable under a regime of dominat temporal incoherence (bunch length of about 3.0 fs). For a further check of the effect of the charge fluctuations with respect to the ones due to the bunch length, we compared the case $\frac{\Delta \sigma }{\sigma }=5\%$ and $\frac{\Delta N}{N}=5\%$ with the homologous case with $\frac{\Delta N}{N}=0\%$ under the scenarios of“coherent spectrum” and “coherent+incoherent spectrum”. The results of this numerical simulation says that the percentage difference between model predictions of $\sigma$ and model-input values stays largely below $1\%$ in the model-input range $\sigma =0.5--2.5$ (fs), whereas it reaches the level of $3\%$ for a model-input value of $\sigma =3.0$ (fs).

The effect of the electronic noise of the ECOL-BCM detectors is only relevant under a regime of a dominant temporal coherence for a bunch-length smaller than 1.0 fs. In particular, the effect of the electronic noise becomes more robust in the dominant temporal coherent regime when $\frac{\Delta \sigma }{\sigma }$ decreases from $5\%$ to $2\%$ (rms). This behaviour sounds quite reasonable since, for a $\frac{\Delta \sigma }{\sigma }$ ranging from from $5\%$ to $2\%$, the percentage weight of the noise-to-signal ratio—$0.5\%$ and $1.0\%$—becomes comparable with the one of $\frac{\Delta \sigma }{\sigma }$. It is worth noting that percentage values of $0.5\%$ and $1.0\%$ for the noise-to-signal ratio are most likely overestimated if compared with a percentage of $2\%$ for $\frac{\Delta \sigma }{\sigma }$ and $\frac{\Delta N}{N}$.

In conclusion, the outcomes of the numerical simulations shown in Figs. 7 and 8 can be summarized as follows.

1. The model prediction of the bunch length is in practice invariant whether the scenario of “coherent spectrum” or “coherent+incoherent spectrum” is supposed for the response signals of the ECOL-BCM.
2. For both the aforementioned scenarios, the agreement of the model predictions with the model input values of the electron bunch length is excellent in the region of the optimal bunch-length sensitivity of the ECOL-BCM and likewise remains under the so called regime of dominant temporal coherence (sub-fs bunch length).
3. For both the aforementioned scenarios, the model prediction of the bunch length starts diverging from the model-input values when the so called regime of dominant temporal incoherence is reached. As it is also evident in the form factors plots of Fig. 6, for a bunch length approaching the threshold of 3 fs, the imprinting of the temporal coherence on the detector signal tends to be smoothed down. For the same reason, the bunch-length prediction of the proposed method is reasonably expected to suffer from a performance degradation as much as the bunch length gets longer and the BCM detector signal becomes weaker.
4. For both the aforementioned scenarios, the relative statistical fluctuations of the bunch charge have a negligible effect on the model prediction of the bunch length compared to the role played by the relative statistical fluctuations of the bunch length. This occurs even in the region of a dominant temporal coherence where, because of the dependency of the detector signal on the square of the bunch charge, the contribution of the bunch length variation to the detector signal may be expected to be less relevant. Evidently, for reasonable values of the variations of the bunch charge and length, even under a regime of temporal coherence, the bunch-length dependent Gaussian modulation of the detector signal is still predominant with respect to the quadratic contribution of the charge. As a crosscheck of this statement, a comparison of the model-predicted results for the bunch length has been carried out under the hypothesis of relative charge fluctuations of $5\%$ and $0\%$ for both scenarios of “coherent spectrum” and “coherent+incoherent spectrum”.
5. Finally, the effect of the electronic noise of the detector in the bunch length sensitivity region of the ECOL-BCM. In the case of the ECOL-BCM, such effect appears observable only under a regime of dominant temporal coherence where the signal variations due to the bunch length variation start being smoothed down, i.e., below 1 fs. In the present sensitivity study, it should be noted that the guess of electronic noise levels of $0.5\%$ and $1.0\%$ is probably extreme if compared with the variation levels—$2\%$ and $5\%$ - supposed for bunch length and charge. In this sense, the numerical simulations performed under the scenario “coherent+incoherent+noise spectrum” can be considered as a stress test of the proposed model under extreme conditions. In conclusion, for the considered case of the ECOL-BCM and under the validity constraint of the assumptions done on the variation levels of the parameters involved in the numerical simulation (charge, bunch length and electronic noise), according to the results plotted in Figs. 7 and 8 the effect of the electronic noise of the detector is negligible in the bunch length interval—$1.0-2.5$ fs (rms)—which can be considered as the optimal sensitivity region of the ECOL-BCM.

## Discussion

The mathematical model we presented allows for the decoding of the individual and independent contributions of the bunch charge and length to the signal of a BCM. By means of the shot-sequential processing of the relative statistical fluctuations of a BCM and a charge monitor signals, such a method allows for the shot-to-shot tracking of the relative variations of the bunch length $\frac{\Delta \sigma }{\sigma }$. In the case of a BCM equipped with two detectors simultaneously detecting the radiation pulse in two distinct wavelength bands, the proposed method allows for the absolute determination of the electron bunch length. Relevant feature of the proposed method is the processing of the relative statistical fluctuations of the BCM and charge signals with respect to the corresponding reference values, for instance, the mean values at the steady state regime of the machine. The model predictions of the absolute value of the electron bunch length are hence less sensitive to possible bunch-length independent but frequency-dependent modulation factors affecting the BCM signal. Indeed, thanks to the processing of the relative variations of the BCM signals, the effects of a possible diffractive low-frequency cut-off of the radiation energy spectrum as well as the effects of a frequency modulation of the detector response can be smoothed down. Low-frequency cut-off of the energy spectrum are due, for instance, to the finite size of the light transfer line or to the finite field extension of the radiating source. In conclusion, the presented results pave the way for a BCM-based fine tuning of the machine compression set-up of a FEL. Indeed, thanks to the proposed analysis method of the BCM signals, the absolute measurement of the electron bunch-length—rather than the simple bunch-length dependent signal of a BCM—can be used to drive the feedback loop of the bunch compression settings of the machine. Finally, for a CW-superconducting-linac driven FEL, where the non-invasiveness is a mandatory constraint for the diagnostics of the machine, the proposed method discloses evident perspectives of a fully non-invasive and absolute determination of the electron bunch length.

## Data Availability

Numerical codes and data are available from the author upon request.
